# NLRP3 Inflammasome Biomarker—Could Be the New Tool for Improved Cardiometabolic Syndrome Outcome

**DOI:** 10.3390/metabo10110448

**Published:** 2020-11-06

**Authors:** Andra-Iulia Suceveanu, Laura Mazilu, Niki Katsiki, Irinel Parepa, Felix Voinea, Anca Pantea-Stoian, Manfredi Rizzo, Florin Botea, Vlad Herlea, Dragos Serban, Adrian-Paul Suceveanu

**Affiliations:** 1 Gastroenterology Department, Ovidius University, 900470 Constanta, Romania; 2 Oncology Department, Ovidius University, 900470 Constanta, Romania; 3 1st Department of Internal Medicine, Diabetes Center, Division of Endocrinology and Metabolism, AHEPA University Hospital, 546 21 Thessaloniki, Greece; nikikatsiki@hotmeil.com; 4 Cardiology Department, Ovidius University, 900470 Constanta, Romania; irinel_parepa@yahoo.com; 5 Urology Department, Ovidius University, 900470 Constanta, Romania; felix.voinea@yahoo.com; 6 Diabetes Mellitus Department, University of Medicine and Pharmacy Carol Davila, 050474 Bucharest, Romania; ancastoian@yahoo.com; 7 Department of Medicine, University of South Carolina, Columbia, SC 29208, USA; manfredi.rizzo@unipa.it; 8 Department of Health Promotion, Mother and Child Care, Internal Medicine and Medical Specialties, University of Palermo, 90133 Palermo, Italy; 9 Liver Transplant and General Surgery Centre, Fundeni Institute, 022328 Bucharest, Romania; boteaflorin@yahoo.com; 10 Department of Pathology, Fundeni Institute, 022328 Bucharest, Romania; herlea2002@yahoo.com; 11 IVth Department of Surgery, University of Medicine and Pharmacy Carol Davila, 050474 Bucharest, Romania; dragos.serban@umfcd.ro; 12 Internal Medicine Department, Ovidius University, 900470 Constanta, Romania; asuceveanu@yahoo.com

**Keywords:** NLRP3 inflammasome, metabolomics, inflammasome, biomarkers, cardiometabolic syndrome, targeted therapy, outcome

## Abstract

Metabolomics, the research area studying chemical processes involving metabolites, finds its utility in inflammasome biomarker discovery, thus representing a novel approach for cardiometabolic syndrome pathogeny acknowledgements. Metabolite biomarkers discovery is expected to improve the disease evolution and outcome. The activation of abundantly expressed NLRP3 inflammasome represents the background process of the diabetes mellitus disturbances like hyperglycemia and insulin resistance, as well as for myocardial cell death and fibrosis, all of them being features characteristic for cardiometabolic syndrome. Many molecules like troponins, brain natriuretic protein (BNP), ST2/IL-33, C-reactive protein (CRP), TNF, IL-1β, and IL-18 cytokines have been already examined as molecular markers for diagnosing or predicting different cardiac disturbances like myocardial infarction, heart failure, or myocarditis. In addition, metabolomics research comes with new findings arguing that NLRP3 inflammasome becomes a promising molecular tool to use for clinical and therapeutical management providing new targets for therapies in cardiometabolic syndrome. Inflammasome markers analyses, along with other molecular or genetic biomarkers, will result in a better understanding of cardiometabolic syndrome pathogenesis and therapeutic targets. Screening, diagnostic, and prognostic biomarkers resulted from inflammasome biomarker research will become standard of care in cardiometabolic syndrome management, their utility becoming the first magnitude.

## 1. Introduction

**Figure 1:**
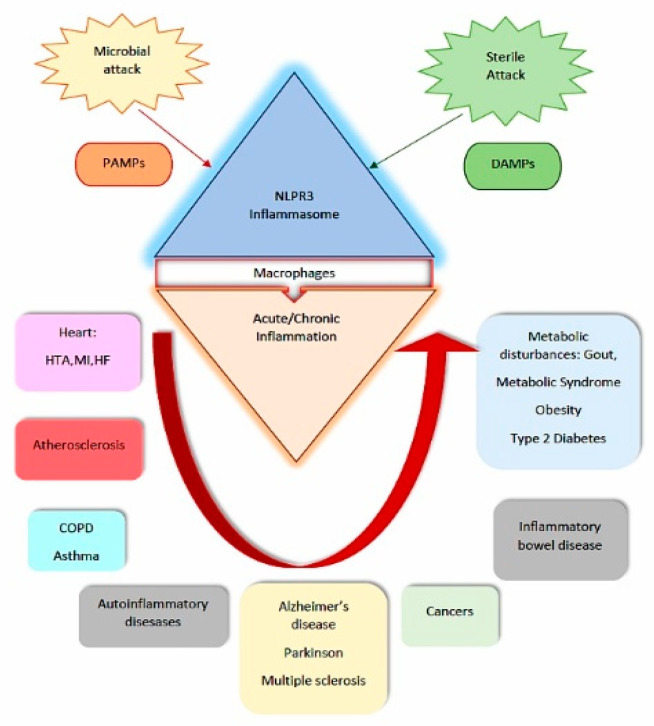
NLPR 3 and its implications in inflammatory diseases. Legend PAMPs = pathogen-associated molecular patterns, DAMPs = damage-associated molecular patterns, HTA = hypertension, MI = myocardial infarction, HF = heart failure, COPD = chronic obstructive pulmonary disease.

Recent literature data provide insight into the inflammation mechanisms that underlie the pathophysiology of cardiometabolic syndrome, including diabetes mellitus (hyperglycemia and insulin resistance), dyslipidemia, obesity along with visceral adiposity, and cardiac impairment [1]. Immune responses and metabolic regulation are two essential mechanisms involved in cellular homeostasis, being responsible for human species survival and conservation. The imbalance between these two mechanisms represents the background of several chronic diseases [2]. Pattern recognition receptors (PRRs) responsible for host immune defense against microbial infection contain different types of proteins, like NOD-like receptors (NLRs), a large multimeric protein complex called the inflammasome, are able to not only recognize microbial signals but also mediate immune responses characteristic for metabolic dysfunctions. Endogenous metabolic danger signals like an amyloid beta in Alzheimer’s disease, calcium pyrophosphate dehydrate (CPPD) crystals in pseudogout, monosodium urate (MSU) crystals in gout, ceramides in obesity, cholesterol crystals in atherosclerosis, islet amyloid peptide (IAPP) or palmitate in T2D trigger the NOD (nucleotide-binding oligomerization domain)-like receptor family (NLR family also called IPAF), of whom the NLRP3 inflammasome components are of best importance. Activation of NLRP3 induces recruitment of ASC (an apoptosis-associated speck-like protein containing a CARD), this molecule being responsible for pro-caspase-1 recruiting and autocatalytic activation of caspase-1. The active caspase-1 converts the inactive pro-IL-1β and pro-IL-18 into active secreting forms. After NLRP3 inflammasome activation, cells secrete large amounts of pro-inflammatory cytokines, which along with the already activated caspase-1 induce cell death, termed “pyroptosis” [3]. Literature evidence emerges that cardiometabolic syndrome (CMS) and its subsidies, obesity, type 2 diabetes (T2D), and cardiovascular disease are based on a common pathogenic background relying on chronic inflammation. On this inflammatory state, the CMS becomes apparent long after its pathogenic onset. Accurate molecular biomarkers called inflammasome markers involving inflammation pathways to become of particular importance since the delay of diagnosis or appliance of preventive measures is of tremendous significance for the disease outcome, profoundly influencing the morbi-mortality rates [4]. The link between different metabolites and NLRP3 inflammasome is lately more actively argued. NLRP3 inflammasome looks to become activated by different substances, like crystalline or other particulate materials. Following the line, researchers proved that cholesterol crystals promote inflammation by activating the inflammasome pathway and this finding raised the horizon for other similar research that demonstrates the link between metabolic diseases like cardiometabolic syndrome and NLRP3 inflammasome biomarkers activation [5]. By targeting inflammation in diabetic patients, physicians become able to improve insulin sensitivity and to ameliorate glucose control, relieving by this approach, the risk of cardiovascular complications. Metabolomics, research area providing inflammasome biomarker discovery, finds in CMS an enormous landscape of practice. Metabolite biomarkers discovery is expected to improve disease evolution and outcome. Inflammasome markers analyses, along with other genetic biomarkers, will allow a better understanding of CMS pathogenesis. Starting with screening biomarkers, for populations at risk for CMS and continuing with diagnostic and prognostic biomarkers, for already diagnosed patients, inflammasome biomarkers become disease surrogates, their importance in the clinical management challenge becoming the first magnitude. NLRP3 component of inflammasome becomes an important component of the inflammatory system involved both in the antibacterial and fungal defense, but also in the pathogeny of chronic diseases that have inflammatory pathogeny as T2D, gout, atherosclerosis, autoimmune diseases, and Alzheimer disease and its specific inhibition may provide the opportunity for targeted therapy in CMS, too [6,7,8]. (Figure 1) On this scenario, anti-IL-1 therapies proved the potential of inhibiting proinflammatory caspase-1, pathogenic step characteristic for CMS occurrence, associated with aberrant inflammasome signaling, raising a new horizon for metabolic diseases management.

This review argues the importance of inflammasome markers becoming targets for future therapeutic and monitoring approaches in CMS.

## 2. Evidence-Based Data on Inflammasome Biomarkers Involvement in CMS Pathogeny

CMS, defined as a heterogeneous group of diseases, poses difficult therapeutic management and lacs of accurate molecular predictors [9,10]. Several biomarkers like C-reactive protein (CRP) [11,12,13], adiponectin [14,15,16], leptin [17,18,19,20,21], were studied as predictors for CMS risk, but results proved their failure as promising biomarkers for CMS prevention or disease prognosis prediction.

The interactions between different genetic and environmental factors are still believed to be the pathogenic backstage for the CMS. Hypercoagulability, oxidative stress, endothelial dysfunction, and inflammation are the main pathogenic verges of the CMS, but the inflammatory process remains of particular interest.

New biomarkers including inflammasome molecules [22,23], cardiac fibrosis markers [24], apelin [25], cytokines [10], metabolites [26], and microRNAs [27,28,29], are still proposed by researchers for determining their biological role in CMS amelioration, diagnosis, or prognosis prediction. As literature data are showing, it appears that no single biomarker, but combinations of biomarkers are more reliable to improve CMS prediction, diagnosis, and prognosis through specific algorithms [22,23,24,25,26,27,28,29,30].

Available literature data also suggest that the presence of abnormal serum inflammatory markers is considered a predictive risk factor for developing T2D and subsidiary cardiovascular disease, especially in overweighed and obese patients. In consequence, in T2D or obese patients, by targeting and ameliorating inflammation, we can reduce the risk of cardiometabolic complications.

Inflammasome activation was considered crucial for the host defense mechanisms to different pathogens. Still, recent data suggest that inflammasomes activation is also essential in the pathogenesis of several diseases having as background the low-grade inflammatory states [30]. A variety of diverse exogenous and endogenous activators including microbial signals, toxins, crystalline substances, peptide aggregates, extracellular ATP released from dying cells may activate NLRP3 assembles. It looks that the component of inflammasomes with interest in CMS pathogenesis is NLRP3 and its ability to recruit the inflammasome-adaptor protein ASC and secondary activation of caspase-1 and caspase-11, which become crucial steps in the process of pro-inflammatory cytokines interleukin (IL)-1β and IL-18 releasing and maturation, and has the ability to initiate apoptosis of cardiomyocytes, activation of cardiofibroblasts, and induction of myocardial fibrosis [31,32,33].

**Figure 2:**
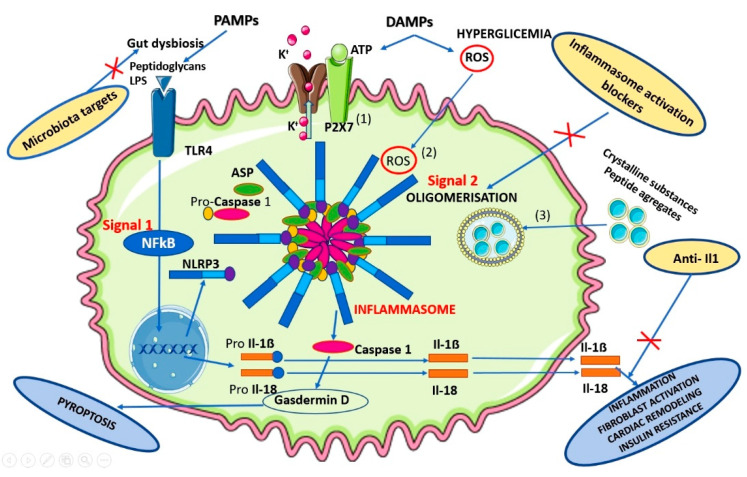
Proposed mechanism of activated NLRP3 in the macrophages exposed to ATP by PAMPs and DAMPs.

Hyperglycemia-induced ROS (reactive oxygen species) overproduction is a major contributor to chronic low-grade inflammation, the activated NLRP3 inflammasome becoming a key in the pathogenesis of metabolic disturbances characteristic for CMS [32,34]. It could be activated in the macrophages by the ample action of pathogen-associated molecular patterns (PAMPs-LPS, peptidoglycans, etc.) or damage-associated molecular patterns (DAMPs) when macrophages are exposed to ATP [32,34]. (Figure 2)

One of the leading causes of death in diabetes patients is the diabetic cardiomyopathy (DCM) [35], which is characterized by both structural and functional myocardial abnormalities. Myocardial cell death, myocardial fibroblast activation, left ventricular dysfunction, and metabolic disturbances are few of the pathogenic features of DCM [36]. The death of cardiomyocyte is thought to initiate cardiac remodeling and results in left ventricular dysfunction [37,38]. Recent metabolomics research proposes a new inflammatory mechanism capable of activating the innate immune system, which contributes to DCM development [39]. The innate immune system represents an ancient part of the immune system responsible for the rapid detection of possible dangers through germline-encoded receptors, capable of recognizing microbial antigens or components resulted from cellular damage—the pattern recognition receptors (PRRs). It seems that the activation of abundantly expressed NLRP3 inflammasome from cardiomyocytes represents the background process of the myocardial cell death. Many molecules like myocyte injury markers troponins I and T, the myocyte stress markers brain natriuretic peptide (BNP) and ST2/IL-33, the inflammation markers C-reactive protein (CRP) and TNF, IL-1β, and IL-18 cytokines [40] have been examined as potential molecular markers for different cardiovascular disease, each of them diagnosing or predicting different cardiac disturbances like myocardial infarction, heart failure, or myocarditis. In addition, metabolomics research comes with new findings arguing that NLRP3 inflammasome becomes a promising molecular marker to use for clinical and therapeutical management providing new targets for therapies CMS.

Moreover, researchers focused on metabolic action of lysophosphatidylcholine (LPC), known as a major bioactive component of oxidized LDL and hypothesize that LPC could be responsible for many of the inflammatory effects described in endothelial cells and represents the motor of the vascular remodeling and secondary atherogenesis. Beside its proinflammatory action on coronary artery smooth muscle cells (CASMCs) trough arachidonic acid releasing activation, LPC stimulates the simultaneous release of fibroblast growth factor, IL-6, IL-8, and GM-CSF cytokines via NLRP3 inflammasome pathways. These findings sustain the hypothesis that LPC might play a multifactorial role in the occurrence and progression of atherosclerosis, by activating the inflammatory processes, including NLRP3 inflammasome pathways [41].

## 3. The New Paradigm of CMS Therapy

The implication of the innate immune system by specific activation of NLRP3 inflammasome pathway is more and more discussed as the pathogenic background of type II diabetes mellitus (T2D), and CMS and its involvement in the therapeutic field changed the paradigm of CMS treatment. It looks like hyperglycemia stimulates human pancreatic β-cells to produce IL-1β, mediating by this way β-cell dysfunction, cell death, worsened hyperglycemia, and inflammatory cycle propagation [42]. New therapeutic molecules targeting links of the above-mentioned mechanism involving IL-1 improve the β-cell secretory function and glycemic control characteristic for T2D.

### 3.1. Targeted Therapies against Specific NLRP3 Inflammasome-Activated Components

In terms of cardiac remodeling and repair, researchers are focused on fibroblasts, myocardial cells, accounting for up to two-thirds of cells in cardiac tissue [43,44]. The NLRP3 inflammasome is the first sensor of DAMPs after myocardial injury, being responsible for the initiation of the inflammatory response in injured cardiac tissue. Fibroblasts activate the NLRP3 inflammasome immediately after myocardial ischemic injury via reactive oxygen species and K+ efflux [45,46]. Fibroblasts are resistant to oxidative stress and are able to initiate a hypoxic immune response by developing an inflammatory and fibrogenic process translated in cytokines activation, inflammatory cell infiltration, myofibroblast transdifferentiation, and increased collagen production [43,45]. ROS and K+ efflux occurred secondary to ischemia, activates the inflammasome in the fibroblast, and activates IL-1β, responsible for the initial immune response. Activated fibroblasts release chemokines that recruit macrophages and neutrophils to the site of insult, continuing and propagating the inflammatory response by releasing further IL-1β and IL-18 cytokines. Fibroblasts play a central role in the initiation and enhancement of myocardial injury after ischemia, and NLRP3 inflammasome activation seems to play a crucial role in the profibrotic gene expression and cardiac fibroblast differentiation [45]. Immune modulating therapies using disease-modifying agents of rheumatoid diseases (DMARDs), such as anakinra and methotrexate, have demonstrated improvements in post-infarction heart failure recently [46]. Anakinra, an IL-1 receptor antagonist used for rheumatoid arthritis treatment [47] and glyburide, a sulfonylurea medication inhibiting NLRP3 inflammasome activation by preventing the secretion of IL-1β [48], are just two drugs sustaining the use of NLRP3 inflammasome-targeted therapies for T2D and CMS. Thioredoxin (TRX)-interacting protein (TXNIP), a protein linked to NLRP3 inflammasome activation, is responsible for insulin resistance and is targeted too by novel therapeutic molecules in clinical trials [49].

Furthermore, a large anti-cytokine study [50] (CANTOS, www.thecantos.org), examined IL-1β inhibition via canakinumab, an IL-1β neutralizing monoclonal antibody, targeting decreased rates of recurrent MI, stroke, and cardiovascular death in patients with coronary artery disease and increased CRP. The researcher’s goal was to provide a novel cytokine-based therapy for the secondary prevention of cardiovascular disease new-onset T2D. Unfortunately, CANTOS trial did not reach the main goal, it did not show expected results, and secondary side effects of canakinumab were substantial in terms of drug-related infections [51]. Still, the CANTOS trial opened new research horizons; the inflammasomes biomarkers study provided tremendous targets for new therapeutic molecules.

The concept of inflammasome-independent NLRP3 implication in fibrosis was also studied in renal tubular cells destroyed in acute kidney injury (AKI). Researchers demonstrated its value as a therapeutic target for the improvement of renal inflammation and fibrosis during AKI in laboratory mice. Their results concluded that inflammasome-independent NLRP3 is involved in mitochondrial ROS production and renal tubular cell injury secondary to hypoxemia, suggesting that inflammasome-independent NLRP3 could be considered as a therapeutic target to prevent the progression of renal disease to a chronic state [52].

In a recent study in Nature Medicine, Youm et al. (2015) [53] reported that the ketone body β-hydroxybutyrate (BHB) specifically inhibits NLRP3 inflammasome activation and the production of active IL-1β and IL-18. This effect was possible by the inhibition of several well-known NLRP3 activators both in mouse bone-marrow-derived macrophages and also in human monocytes.

Researchers found that already known drugs like rosuvastatin (RSV) have anti-inflammatory action expressed by their ability to inhibit the activation of NLRP3 inflammasome through TXNIP pathway. The suppression effect on NLRP3 inflammasome by the phosphorylation of MAPK signal pathways, characteristic for DCM, was described by Luo et al. [37]. The administration of INF4E (a small electrophilic molecule), which has an inhibition effect on NLRP3 inflammasome providing protection against myocardial injury, the activation of the prosurvival RISK pathway, and the improvement in mitochondrial function was described by Mastrocola et al. [54]. These mechanisms are just a few of the rosuvastatin anti-inflammatory effects relying on the inflammasome markers utility and providing arguments for anti-inflammatory targeted therapy in CMS. In the light of discoveries, targeting specific NLRP3 inflammasome inflammatory pathways, researchers may reduce inflammation without affecting other innate host defense mechanisms as classic anti-inflammatory drugs do [55]. Besides, by blocking early inflammatory mediators as NLRP3 inflammasome components induced by hyperglycemia will represent the excellent targets to prevent T2D and its complications like DCM [56].

### 3.2. Microbiota Targets

Glucose, fatty acids, and islet amyloid polypeptide activate the NLRP3 inflammasome driving to metabolic disorders, T2D, and secondary CMS by pancreatic islet inflammation and subsequent *β*-cell failure and destruction [57,58,59]. T2D and CMS are characterized by a dysbiotic state. Gut dysbiosis can contribute to the onset and maintenance of insulin resistance, influencing the prognosis of the metabolic state. Gut microbiota changes are observed both in high-risk patients and diagnosed T2D patients. These changes interest both the composition and the function of the microbiota [60,61]. Metabolites resulted from gut microbiota activity, such as short-chain fatty acids (SCFA) have an important impact on the normal metabolic processes like insulin secretion, incretin production (GLP-1, GIP), and immune system competence. Gut microbiota disturbances like reduced Gram-positive bacteria from Firmicutes spp (Clostridia, Bacilia, etc.,) decreased in butyrate-producing bacteria (Roseburia, Butyrivibrio) and increased Gram-negative spp (E. Coli, Bacteroides) specific for T2D and CMS are responsible for diminishing insulin sensitivity, epithelial dysfunction and increase epithelial permeability, increased capacity for harvesting energy and stimulating lipogenesis and NLRP3 inflammasome activation. The type of food intake and especially the dietary fiber content may have stimulating or inhibitory effects on NLRP3 inflammasome via metabolites such as SCFA produced by microbiota digestion [8,62,63]. Intraluminal food ingestion through the gut flora action is responsible for glucose production and peritoneal macrophages activation, finally resulting in the release of IL-1*β* and insulin secretion [64,65,66,67] by activating the highly expressed IL-1 receptor type 1 on pancreatic β-cells. Furthermore, insulin stimulates macrophage-derived pro–IL-1β maturation via the NLRP3 inflammasome [65]. By the restoration of gut dysbiosis through probiotics, primary bile acids will be metabolized to secondary bile acids, leading to an improvement in insulin sensitivity and decrease inflammation through inhibition of NLRP3 inflammasome. A recent meta-analysis of studies made on laboratory animals published by Carmen Tenorio-Jiménez et al. [68] concludes that probiotics have beneficial effects only in addition to drugs therapy and healthy lifestyle and the benefit is dose- and strain-specific. However, RCTs on humans are needed to elucidate the benefit of probiotics in CMS.

### 3.3. Diet Targets

On the other hand, different constituents of diet play a significant role in NLRP3 activation. Small molecule supplementation could help to manipulate the NLRP3 inflammasome activity. MCC950 is a water-soluble compound that has the ability to inhibit several NLRP3 activators, with no action over signal 1 activation [34] still, its role exerted on sterile inflammation is yet to be demonstrated. PUFAs (polyunsaturated fatty acids) contained in walnuts, sunflower seeds, flax seeds or flax oil, corn oil soybean oil, safflower oil and omega-3 fatty acids found in fish, like salmon, mackerel, herring, albacore tuna, and trout were proved to reduce the negative effects of metabolite-stimulated NLRP3 [69].

## 4. Conclusions

Specific inhibition of NLRP3 inflammasome pathways may give rise to the opportunity for targeted therapies for CMS in which inflammasome activation is the background of pathogenesis events. In this scenario, anti-IL-1 therapies proved to have therapeutic effect in inhibiting proinflammatory caspase-1, pathogenic element characteristic for CMS, and associated with aberrant inflammasome signaling, resulting in a new horizon for metabolic diseases treatment. Other drugs with anti-inflammatory effects targeting the inflammasome biomarkers activated in CMS, such as rosuvastatin or probiotics, are also used with success. Discovering new inflammasome biomarkers has prompted new challenges for researchers, and new drugs-targeting elements might lead to beneficial effects for CMS outcome. This new approach will help clinicians to not only predict the onset of cardiometabolic syndrome but also the disease outcome.
